# Molecular characteristics of circulating tumor cells resemble the liver metastasis more closely than the primary tumor in metastatic colorectal cancer

**DOI:** 10.18632/oncotarget.10175

**Published:** 2016-06-20

**Authors:** Wendy Onstenk, Anieta M. Sieuwerts, Bianca Mostert, Zarina Lalmahomed, Joan B. Bolt-de Vries, Anne van Galen, Marcel Smid, Jaco Kraan, Mai Van, Vanja de Weerd, Raquel Ramírez-Moreno, Katharina Biermann, Cornelis Verhoef, Dirk J. Grünhagen, Jan N.M. IJzermans, Jan W. Gratama, John W.M. Martens, John A. Foekens, Stefan Sleijfer

**Affiliations:** ^1^ Erasmus MC Cancer Institute, Department of Medical Oncology and Cancer Genomics Netherlands, Rotterdam, The Netherlands; ^2^ Department of Surgery, Erasmus University Medical Center, Rotterdam, The Netherlands; ^3^ Department of Pathology, Erasmus University Medical Center, Rotterdam, The Netherlands

**Keywords:** circulating tumor cells, CTCs, CellSearch, colorectal cancer, gene expression profiling

## Abstract

**Background:**

CTCs are a promising alternative for metastatic tissue biopsies for use in precision medicine approaches. We investigated to what extent the molecular characteristics of circulating tumor cells (CTCs) resemble the liver metastasis and/or the primary tumor from patients with metastatic colorectal cancer (mCRC).

**Results:**

The CTC profiles were concordant with the liver metastasis in 17/23 patients (74%) and with the primary tumor in 13 patients (57%). The CTCs better resembled the liver metastasis in 13 patients (57%), and the primary tumor in five patients (22%). The strength of the correlations was not associated with clinical parameters. Nine genes (*CDH1*, *CDH17*, *CDX1*, *CEACAM5*, *FABP1*, *FCGBP*, *IGFBP3*, *IGFBP4*, and *MAPT*) displayed significant differential expressions, all of which were downregulated, in CTCs compared to the tissues in the 23 patients.

**Patients and Methods:**

Patients were retrospectively selected from a prospective study. Using the CellSearch System, CTCs were enumerated and isolated just prior to liver metastasectomy. A panel of 25 CTC-specific genes was measured by RT-qPCR in matching CTCs, primary tumors, and liver metastases. Spearman correlation coefficients were calculated and considered as continuous variables with r=1 representing absolute concordance and r= -1 representing absolute discordance. A cut-off of r>0.1 was applied in order to consider profiles to be concordant.

**Conclusions:**

In the majority of the patients, CTCs reflected the molecular characteristics of metastatic cells better than the primary tumors. Genes involved in cell adhesion and epithelial-to-mesenchymal transition were downregulated in the CTCs. Our results support the use of CTC characterization as a liquid biopsy for precision medicine.

## INTRODUCTION

The treatment of metastatic colorectal cancer (mCRC) increasingly depends on the tumor's molecular characteristics. For example, inhibition of the Epidermal Growth Factor Receptor (EGFR) by cetuximab or panitumumab was shown to be futile in the 30-60% of mCRC patients with *KRAS* or *NRAS* mutated tumors, and as such, these treatments are now indicated only for patients with wild-type tumors [1, 2]. Other tumor cell characteristics besides gene mutations may further affect patient outcome, as evidenced by a recent study showing the ability of a gene expression profile to predict outcome to chemotherapy in mCRC patients [3]. One may argue that treatment decisions are best based on the composite picture of several molecular features, including DNA mutations and transcription levels.

Blood sampling for circulating tumor cells (CTCs) has widely been proposed as a “liquid biopsy” to guide treatment decisions. In addition to the CTC count, which is strongly prognostic for survival in patients with mCRC as determined by the CellSearch System (Janssen Diagnostics, Raritan, NJ) [4], CTCs are generally thought to provide a real-time picture of different tumor characteristics, including the extent of heterogeneity at specific moments [5]. However, solid proof that CTCs can indeed function as surrogates for metastatic tissue is currently lacking, since research on the biology and predictive value of CTCs is hampered by technical difficulties. The characterization of CTCs is very challenging due to the rarity of CTCs in the circulation and the large background of leukocytes in which they are left even after CellSearch enrichment [6–9]. In this study, we used our previously described approach to reliably measure the expression of tumor-associated genes in CellSearch-enriched CTCs to compare the molecular characteristics of CTCs with the primary tumor and a liver metastasis from patients with mCRC. We investigated whether the characteristics of CTCs taken at the time of metastatic disease were closer to the liver metastasis or the primary tumor and, in this respect, whether or not we can use CTCs as surrogates for metastatic tissue biopsies.

## RESULTS

**Figure 1:**
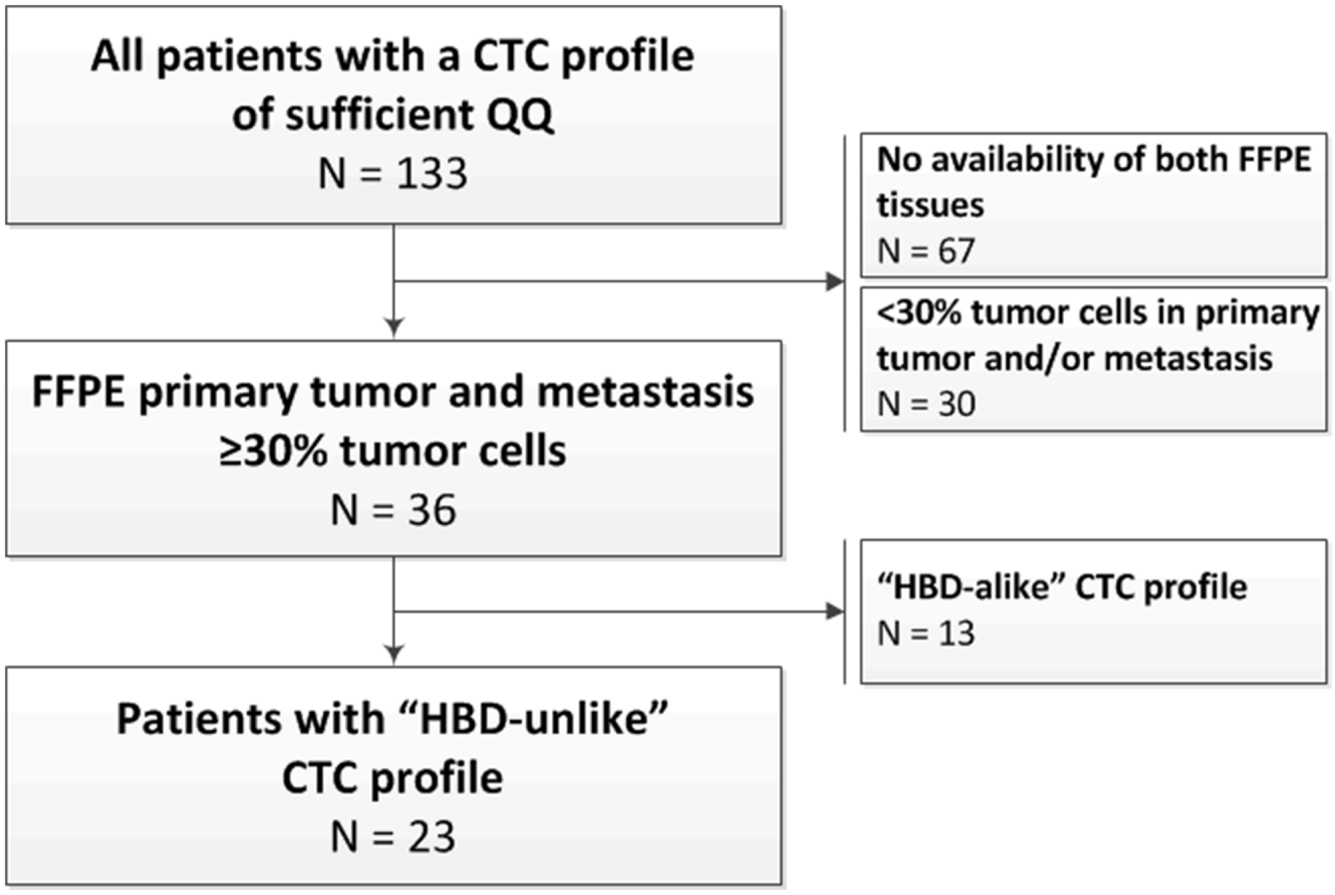
Study flowchart and the selection of patients for the analyses The selection of patients with a gene expression profile from the CTCs, the primary tumor, and the liver metastasis was further based on the presence of sufficient epithelial signals in the CTC samples, as a measure for the presence of CTCs amongst the leukocytes (also see Figure 2). Of the 36 patients, 23 were designated as having an “HBD”-unlike and reliably CTC-driven profile. These patients were included in the analyses to compare the gene expression profiles of the CTCs to the primary tumors and the liver metastases.

**Figure 2:**
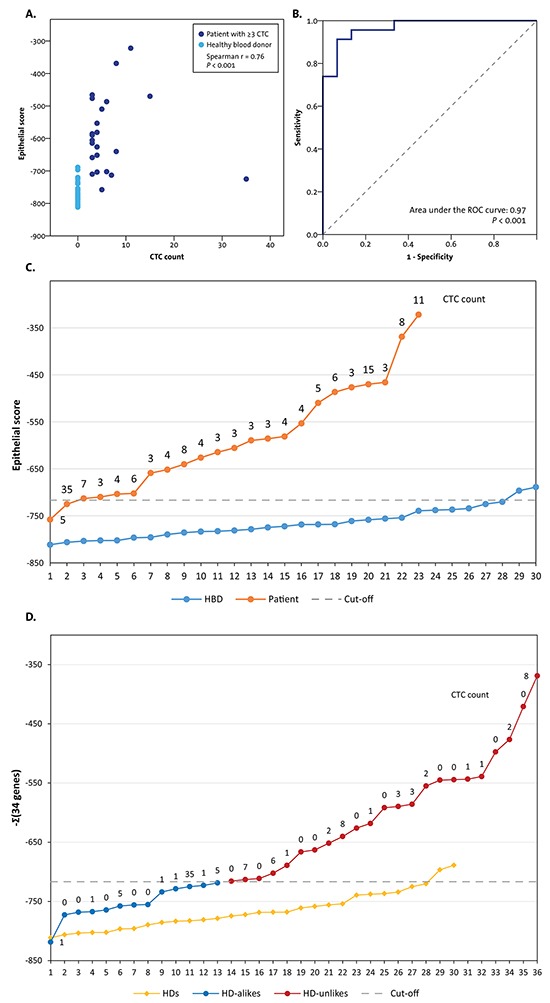
The selection of patients with CTC-driven profiles from the blood samples of the total 36 selected patients Only patients with sufficient epithelial input were included in the analyses to compare gene expression profiles with CTCs, the primary tumor, and a liver metastasis. **A.** An epithelial score was calculated by adding the expression levels of the 34 CTC-specific genes multiplied by the *z*-value from the comparison between 23 patients with ≥3 CTCs and using the 30 HBDs from the prior study [9] as a weighing factor. The epithelial scores from the 23 patients with ≥3 CTCs and the 30 HBDs strongly correlated with the CTC count from the blood tube taken in parallel with the tubes for the characterization of CTCs (r=0.76, *P*<0.001). **B.** A Receiver Operating Characteristics (ROC) curve was constructed from the epithelial scores of the 23 patients with ≥3 CTCs and the 30 HBDs. The optimal cut-off value resulted in a sensitivity of 91% and a specificity of 93% to discriminate patients from HBDs. **C.** Line graph showing the epithelial scores of the 23 patients with ≥3 CTCs and the 30 HBDs. The dashed line shows the optimal cut-off value from the ROC curve. Two patients were assigned as HBDs, one of whom had a CTC count of 35. Most probably this is the result of a technical error in the enrichment of the CTCs or the gene profiling. Two HBDs had an epithelial score slightly above the cut-off value and were assigned as patients. **D.** The epithelial scores were calculated for the patients selected for the current study with FFPE primary tumors and liver metastases. Of the 36 patients, 23 had a score above the cut-off and were designated as having an “HBD”-unlike profile. These patients were included in the analyses to compare the gene expression profiles of the CTCs to the primary tumors and the liver metastases.

A total of 142 patients were included in the original prospective study investigating the prognostic value of the CTC count [10]. Archived formalin-fixed paraffin-embedded (FFPE) primary tumor and liver metastasis tissues with ≥30% tumor cells on haematoxylin and eosin (HE) slides were available from 36 patients (Figure 1). Only patients with truly CTC-driven profiles from the CellSearch-enriched peripheral blood were selected for the comparison of the CTC profiles with the primary tumor and liver metastasis profiles. To this end, we calculated epithelial scores from the CTC samples as an indication for tumor cell input. Samples with an epithelial score above the established cut-off were selected (see methods section and Figure 2A-2D). The epithelial score was below the cut-off in 13 patients, leaving 23 patients with a reliable CTC-driven gene expression profile suitable for comparison with the primary tumor and liver metastasis (Figure 2D). The characteristics of these patients are shown in Table 1.

**Table 1:** Clinicopathological characteristics of the 23 patients with “HBD-unlike” profiles

	*N*	%^*^
**Total**	23	100%
**Age at inclusion** (mean ± sd)	68 ± 10	
**Sex** (Male / female)	16 / 7	70% / 30%
**Location primary tumor**		
Right hemicolon	6	26%
Left hemicolon / sigmoid	12	52%
Rectum	5	22%
**Staging**		
T2	3	13%
T3	16	70%
T4	2	9%
Unknown	2	9%
N0	9	39%
N1-2	11	49%
Unknown	3	11%
**Differentiation**		
Well differentiated	1	4%
Moderately differentiated	15	65%
Poorly differentiated	1	4%
Unknown	6	26%
**Presentation with metastases**		
Synchronous	12	52%
Metachronous	11	48%
Median interval (IQR)	25 (17 – 39)	
**Liver metastases only**	21	91%
**Dukes classification at first diagnosis**		
A	1	4%
B	4	17%
C	5	22%
D	12	52%
Unknown	1	4%
**Prior chemotherapy**		
Neoadjuvant	1	4%
Adjuvant	3	11%
Induction	7	30%
**Primary tumor *in situ* at CTC draw**	4	17%
**Number of CTCs before liver surgery** (median, IQR)	1 (0-3)	
≥3 CTCs	6	26%

* Percentages do not always add up to 100% due to rounding

IQR = interquartile range; sd = standard deviation.

**Figure 3:**
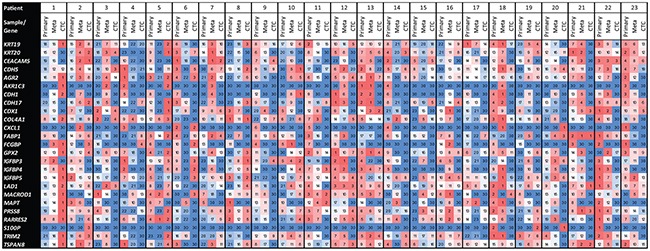
Heatmaps showing the ranks per gene, per sample, per patient Expression levels for individual genes have been ranked per sample over the 23 patients; undetectable expression levels have been given a rank number of 30. Red represents higher than median gene expression levels, white represents the median gene expression, and blue represents expression levels below the median or wholly undetectable.

To compare the concordance of the three profiles per patient, heatmaps were constructed to show the relative height of the expression levels per gene in the different tumor compartments. Spearman correlation coefficients over the 25 ranks were calculated and considered as continuous variables with r=1 representing absolute concordance and r= -1 representing absolute discordance (Figure 3; Table 2). With a cut-off of r>0.1, the CTC profiles were concordant with the liver metastasis in 17 patients (74%) and with the primary tumor in 13 patients (57%). The primary tumor and metastasis profiles were concordant in 16 of the 23 patients (70%). Comparing the correlation coefficients from the correlation between the CTC versus primary tumor profiles and the CTC versus liver metastasis profiles with an error margin of Δr>0.1, the CTCs more closely resembled the metastasis in 13 patients (57%) and the primary tumor in five patients (22%; Table 2). In the remaining five patients, the Δr was ≤0.1 and/or both coefficients were ≤0.1. In patients 1 and 20, the CTCs neither resembled the primary tumor nor the liver metastasis. In patients 9, 14, and 17, both correlations seemed similar and the CTCs seemed to reflect both the characteristics from the primary tumor as well as the liver metastasis.

**Table 2:** Correlation coefficients from Spearman correlation analyses comparing the ranked 25 gene profiles from the CTCs, the primary tumor, and the liver metastasis per patient

Patient	Spearman r			CTCs closest to	Clinical parameters				
	PT-CTC	M-CTC	PT-M		CTC count	PT *in situ*	Prior chemo	Presentation with M	Number of M
1	0.08	0.08	0.55	Neither	0	N	N	Synchr	3
2	−0.18	0.12	−0.13	M	2	N	Y	Metachr	1
3	0.17	0.32	−0.21	M	0	N	Y	Synchr	1
4	−0.41	0.17	0.15	M	7	N	Y	Metachr	1
5	0.05	0.12	0.50	M	8	N	Y	Synchr	1
6	0.23	−0.45	0.01	PT	1	N	N	Synchr	1
7	0.33	0.43	−0.10	M	1	N	N	Metachr	1
8	0.24	0.37	0.42	M	0	Y	N	Synchr	2
9	0.20	0.21	−0.01	Both	0	N	N	Metachr	1
10	−0.11	0.28	0.26	M	0	Y	N	Synchr	1
11	0.13	0.42	0.43	M	0	Y	N	Synchr	2
12	0.13	0.03	0.54	PT	0	N	Y	Synchr	7
13	0.05	0.43	0.55	M	2	N	Y	Synchr	2
14	0.15	0.12	−0.38	Both	0	N	Y	Metachr	2
15	0.58	0.30	0.14	PT	8	N	N	Metachr	1
16	0.15	−0.09	0.77	PT	3	N	N	Metachr	1
17	0.25	0.31	0.59	Both	2	N	N	Synchr	2+
18	0.19	0.08	0.12	PT	0	Y	Y	Synchr	4+
19	−0.14	0.13	0.16	M	1	Y	N	Synchr	1
20	−0.15	0.04	0.16	Neither	0	N	N	Metachr	1
21	0.33	0.44	0.58	M	3	N	N	Metachr	2
22	−0.02	0.35	0.16	M	6	N	Y	Metachr	>10
23	0.06	0.56	−0.06	M	1	N	Y	Metachr	3

The cut-off value of r>0.1 was used to consider two profiles concordant. To assess whether a CTC profile was closer to the liver metastasis than to the primary tumor the difference between the correlation coefficients of the CTCs versus the primary tumor and the CTCs versus the liver metastasis had to be >0.1. The clinical parameters tested for the associations with the strength of correlation have been specified per patient. CTC= circulating tumor cells; M = metastasis; PT = primary tumor; Synchr = synchronous; Metachr = metachronous.

We next examined whether clinicopathological parameters were associated with the strength of the correlations. The primary tumor was still *in situ* at the time of liver surgery and CTC sampling in five patients (Table 2). Here, the CTCs could be theoretically derived from both the primary tumor and the metastases. In two patients, the CTCs seemed to share characteristics with both the primary tumor and the liver metastasis, as defined by a positive correlation of r>0.1 with both the primary tumor and the liver metastasis. In patients 10 and 19, the CTCs correlated with the liver metastasis only, whereas in patient 18, the CTC characteristics correlated with the primary tumor only. No associations of the correlations' strength were observed regarding time or pattern of presentation with metastasis, the number of metastases, prior chemotherapy, or age (Table 3).

**Table 3:** Associations between clinical parameters and the strength of the correlation between two tumor samples (CTCs versus primary tumor, CTCs versus liver metastasis, or liver metastasis versus primary tumor)

	*N*	CTC-PT		CTC-M		M-PT	
		Mean r	*P*	Mean r	*P*	Mean r	*P*
Mean all patients	23	0.10		0.21		0.23	
Synchronically metastasized	11	0.11	0.90	0.18	0.50	0.33	0.12
Metachronically metastasized	12	0.09		0.24		0.12	
Solitary metastasis	12	0.06	0.33	0.13	0.10	0.14	0.18
Multiple metastases	11	0.14		0.29		0.32	
Mean primary tumor *in situ*	5	0.06	0.60	0.26	0.47	0.28	0.47
Mean primary tumor resected	18	0.11		0.19		0.21	
Prior chemotherapy received	10	0.02	0.10	0.23	0.69	0.12	0.17
No chemotherapy received	13	0.16		0.19		0.30	
Linear correlations							
Age	23	0.27	0.22	−0.02	0.94	−0.15	0.49
Interval between surgery for PT and M*	12	0.16	0.61	0.24	0.45	−0.44	0.15

For the categorical variables, the reported r values are the mean correlation coefficients from the Spearman rank correlation of the 25 gene profiles. The *P* values are from independent samples *t* tests. For the continuous variables of age and interval between the two surgeries, the reported r and *P*-values are from linear correlations between the variables and correlation coefficients from the 25 gene profiles. CTC= circulating tumor cells; M = metastasis; PT = primary tumor.

Lastly, we investigated the 25 individual genes for differences in expression levels between the three tumor compartments. For this, we calculated the difference between the ranks of two samples (Δrank) per gene per patient and the mean of the Δranks over the 23 patients. This resulted in three mean Δranks per gene (CTC-primary tumor, CTC-metastasis, metastasis-primary tumor; Table 4). In an instance where a gene was not differentially expressed between two tumor compartments, the mean Δrank would be close to and not statistically significantly different from zero. A one-sample t test against 0 was applied to determine whether genes were significantly over- or under-expressed (Table 4). The expression levels between the primary tumor and the liver metastases were overall similar; only *FCGBP* was downregulated in the liver metastases. In the CTCs, however, a larger number of genes was downregulated. In comparison to the primary tumor, the expression of *CDH1*, *CDH17*, *CDX1*, *CEACAM5*, *FABP1*, *FCGBP*, *IGFBP3*, *IGFBP4*, and *MAPT* were downregulated. Compared to the liver metastases, downregulations of the same genes were observed, with the exceptions of *FCGBP* and *IGFBP4*.

**Table 4:** List of the 34 genes that made up our “CTC-specific” gene panel that proved to be reliably measurable in CTCs in a background of leukocytes [8]

ID	Gene Name	Included in final panel?	CTC-PT		CTC-M		M-PT	
			Mean Δrank	*P*	Mean Δrank	*P*	Mean Δrank	*P*
1	*AGR2*	Yes	−2.39	0.43	−2.09	0.45	−0.30	0.90
2	*AKR1C3*	Yes	−2.30	0.45	−3.39	0.29	1.09	0.73
3	*CD44*	No^*^						
4	*CDH1*	Yes	−10.52	**0.02**	−11.26	**0.001**	0.74	0.85
5	*CDH17*	Yes	−8.17	**0.03**	−7.91	**0.05**	−0.26	0.89
6	*CDH5*	Yes	1.48	0.61	1.04	0.66	0.43	0.88
7	*CDX1*	Yes	−11.09	**0.001**	−11.13	**0.004**	0.04	0.98
8	*CEACAM5*	Yes	−11.09	**0.002**	−11.17	**0.004**	0.09	0.97
9	*COL4A1*	Yes	−3.00	0.21	−3.04	0.32	0.04	0.98
10	*CXCL1*	Yes	−4.43	0.12	0.00	1.00	−4.43	0.19
11	*EGFR*	No^*^						
12	*FABP1*	Yes	−7.35	**0.02**	−7.35	**0.02**	0.00	1.00
13	*FCGBP*	Yes	−11.26	**0.02**	1.13	0.68	**-12.39**	**0.004**
14	*GPX2*	Yes	−0.96	0.75	−1.78	0.48	0.83	0.76
15	*HOXB9*	No^*^						
16	*IGFBP3*	Yes	−11.09	**0.003**	−11.09	**0.002**	0.00	1.00
17	*IGFBP4*	Yes	−7.61	**0.02**	−6.43	0.08	−1.17	0.73
18	*IGFBP5*	Yes	−1.00	0.65	−1.00	0.63	0.00	1.00
19	*KRT19*	Yes	−1.09	0.70	−1.09	0.65	0.00	1.00
20	*KRT20*	Yes	−4.26	0.14	−3.61	0.23	−0.65	0.75
21	*KRT8*	No^*^						
22	*LAD1*	Yes	−2.48	0.38	−2.48	0.22	0.00	1.00
23	*MACROD1*	Yes	−1.30	0.72	−2.35	0.45	1.04	0.72
24	*MAPT*	Yes	−14.48	**0.001**	−12.52	**0.003**	−1.96	0.52
25	*NQO1*	No^*^						
26	*PRSS8*	Yes	−1.52	0.54	−1.52	0.51	0.00	1.00
27	*RARRES2*	Yes	−5.00	0.10	−5.00	0.11	0.00	1.00
28	*REG1A*	No^*^						
29	*S100A16*	No^*^						
30	*S100P*	Yes	1.17	0.68	3.65	0.17	−2.48	0.06
31	*SATB2*	No^*^						
32	*SLC6A8*	No^*^						
33	*TRIM2*	Yes	−5.70	0.08	−5.70	0.06	0.00	1.00
34	*TSPAN8*	Yes	−1.96	0.48	−1.30	0.57	−0.65	0.81

* Performed poorly on FFPE tissues in the multiplexed RT-qPCR (linear correlation, r<0.7 and *P*>0.05).

To allow for comparison between the FF CTC samples and the FFPE tumor samples, all Taqman assays were tested on matching FF and FFPE primary tumors from 15 patients. Only genes with correlating expression levels in the matching tissues (linear correlation r>0.7 and *P*<0.05) were included in the final gene panel. In total, 25 of the 34 genes were deemed reliably measurable in all samples and tissues and these genes were used to compare the characteristics of the CTCs to the corresponding FFPE primary tumor and liver metastasis. All individual gene expression levels were ranked over the 23 patients per sample and Δranks of one gene between two corresponding samples from a patient were calculated. The mean Δranks for the 25 genes across the 23 patients are shown in columns 4 (mean difference between the CTCs and the primary tumors), 6 (mean difference between the CTCs and the liver metastases), and 8 (mean difference between the primary tumors and the liver metastases). The mean Δranks were then tested by one-sample *t* tests with 1,000k bootstrapping against the 0 value; the resulting *P* values can be found in the columns 5, 7, and 9. Where there was no significant difference in the average expression of a gene between two samples, the mean Δrank would be close to and not statistically significantly different from 0. CTC= circulating tumor cells; M = metastasis; PT = primary tumor.

## DISCUSSION

In this study, we observed that the molecular characteristics of CTCs obtained just prior to liver metastasectomy well reflected the characteristics of (one of) the liver metastasis and were generally closer to the metastasis than the primary tumor in patients with mCRC. Based on the expression of 25 CTC-specific and tumor-associated genes, we found the CTC profiles to correlate with the liver metastasis in 74% of the patients and with the primary tumor in 57% of the patients. No associations were observed between the strength of the correlations and clinicopathological characteristics.

To gain insight into the molecular changes occurring during tumor progression, we investigated the differences in the expression levels of the 25 individual genes between the three tumor compartments. Nine genes were downregulated in the CTCs, three of which (*CDH1*, *CDH17*, *CEACAM5*) are involved in cell adhesion. Downregulation of *CDH1*, encoding E-Cadherin, is a well-recognized event in the progression of epithelial cancers and the induction of epithelial-to-mesenchymal transition (EMT) [14, 15]. The loss of epithelial markers, including E-cadherin, together with an overexpression of mesenchymal markers has been consistently observed in CTCs and is thought to reflect EMT as a means for CTCs to survive in the circulation [16–19]. Downregulation of insulin growth factor binding proteins 3 (*IGFBP3*) and 4 (*IGFBP4)*, both proliferation-inhibiting and apoptosis-inducing factors, may help CTCs to survive [20]. Additionally, *IGFBP3/4* may play a role in EMT through interactions with the EMT-inducer transforming growth factor β (TGF-β) [20, 21]. The significance of the downregulation of *CDX1*, *FABP1*, and *MAPT* in CTCs is unknown, although associations between the losses of these genes and the development and progression of colon cancer have been described [22–29]. Altogether, most of the downregulated genes in the CTCs seem to act as tumor suppressors, cell adhesion molecules, or have an involvement in EMT, a process that has well-acknowledged relevance for the survival and dissemination of CTCs [14, 15]. The observed downregulations thus seem to have a functional role in CTC biology.

Several studies have compared the characteristics of CTCs to the primary tumors in different solid tumors, including mCRC. For example, mutations in the *KRAS* oncogene were found to be discordant between CTCs and primary tumors from mCRC patients in 6-55% of patients [30–34]. This discordance has been interpreted as tumor heterogeneity and a reflection of the characteristics of metastatic lesions instead of the primary tumor by the CTCs. However, solid proof that CTCs can indeed function as surrogates for metastatic tumor cells and thus prove to be a reliable alternative for tissue biopsies is lacking. Few studies have made direct comparisons between CTCs, the primary tumor, and distant metastatic tissue. In a study on metastatic breast cancer, the expression of the estrogen receptor was concordant between the CTCs and the primary tumor in 15 of the 22 (68%) patients and between the CTCs and the metastases in 10 of the 12 (83%) patients [35]. Notably, in the two patients where the metastasis was discordant from the primary tumor, the CTCs reflected the characteristics of the metastasis. In mCRC, the profiles from single CTCs – obtained with a micromanipulator after CellSearch-enrichment, followed by whole genome amplification, array comparative genomic hybridization and ultradeep sequencing – were compared to the primary tumors and distant metastatic sites of three patients [36]. In one patient, the copy number profile of a single CTC was 73% concordant with the liver metastasis, and 70% with the primary tumor. In the second patient, the CTCs were much closer to the primary tumor, while in the third patient all three profiles closely matched. These results seem comparable to the results from our study in that they support the hypothesis that CTCs are representative for metastatic tissue.

Still, our analyses should be considered exploratory since formal statistical analyses were restricted by the sample size and lack of preliminary data needed for upfront power calculations. Technical issues – mainly caused by the rarity of CTCs in the blood stream and the leukocyte contamination even after CellSearch enrichment – limited the number of genes that could be measured and compared. Nevertheless, we were able to build a CTC-specific gene panel through selection of mCRC-associated genes from literature and testing for absent or low-level expression in leukocytes. Tumor heterogeneity and sampling bias could also be an influence on the results. Only one liver metastasis was profiled per patient, even from patients in whom multiple metastases were present. The number of CTCs that were detected was low and, due to stochastic variations, only a subset of CTCs from the total circulating CTC pool may have been interrogated. Furthermore, the biological behaviors of tumor subclones may differ, whereby smaller, but more aggressive clones may shed more CTCs than an abundant, but more indolent clone, which might be overrepresented in a tissue biopsy. To address the aforementioned issues, future studies should incorporate more extensive sampling of tumor tissues and compare the profiles to single CTC profiles, preferably though an RNA sequencing approach to gain better insight into oncogenic and mutagenic genes and pathways.

In conclusion, CTCs from the majority of patients with mCRC reflected the characteristics of the liver metastasis, supporting the use of CTCs as a surrogate for metastatic biopsies. The CTCs, overall, resembled the molecular characteristics of the liver metastasis better than the primary tumor. Several CTC-specific changes occurred and seemed to primarily represent EMT-related downregulations of cell-adhesion and tumor suppressor genes, which could have a biological function for CTC survival and migration. Our results support the hypothesis that CTCs may become a valuable tool for precision medicine by functioning as a liquid biopsy and providing real-time information on tumor characteristics.

## MATERIALS AND METHODS

### Patients

Patients were retrospectively selected from a previously reported prospective clinical trial investigating the prognostic value of CTC enumeration for the one-year recurrence rate in patients with mCRC undergoing a liver metastasectomy [10]. The selection of patients for the current study is shown in Figure 1. The Erasmus MC Review Board approved the study (METC 06-089). All patients provided written informed consent.

### Sample collection and processing

Archived FFPE primary tumors and liver metastases were collected from pathology laboratories. The High-Pure RNA Paraffin Kit (Roche Applied Science, Penzberg, Germany) was used according to the manufacturer's instructions to isolate RNA from tumors with ≥30% tumor cells on HE staining. The details of blood sampling and processing for the CTC enumeration and characterization have been described before [10, 11]. In brief, two samples of 30 mL blood in CellSave (Janssen Diagnostics, Raritan, NJ) and EDTA tubes were taken just prior to liver surgery and processed <24 h using the CellSearch System. The higher volume of blood used to enumerate CTCs from when compared to the usual 7.5 mL was part of the design of the original study and has been described before [11]. After a modified Ficoll density-gradient separation, mononuclear cells were collected and processed by the CellSearch System using the Epithelial Cell Kit for the CTC enumeration and the Profile Kit for the CTC isolation (both kits Janssen Diagnostics, Raritan, NJ). The isolation of mRNA from CTC samples was performed using the AllPrep DNA/RNA Micro Kit (Qiagen, Venlo, The Netherlands).

The gene expression profiles from all the CTC samples from all patients included in the prospective trial were determined in our previous study [9]. A panel of 34 CTC-specific genes was identified and proved to be reliably measurable in CTCs in the background of leukocytes. The genes had been selected based on literature for their association with mCRC development and progression. They were tested for absent or low-level expression in leukocytes, thereby rendering them measurable in the few CTCs present in the CellSearch-enriched samples. For the current study, we used the same panel of 34 genes for the selected primary tumor and liver metastasis tissues. The Taqman-based RT-qPCR assays used on the CTC samples were tested for performance on FFPE tumor tissue by comparing a separate group of 15 patient-matched fresh-frozen (FF) and FFPE tumor tissues. Only assays with significantly correlating expression levels (linear correlation r>0.7, *P*<0.05) were included in the final gene panel, which resulted in 25 of the 34 genes suitable for use in the comparison of the CTC, primary tumor, and metastasis profiles (Table 4).

Next, we selected patients with truly CTC-driven profiles from the total of 36 with available tissue profiles. Stochastic variations occurring in small numbers, such as CTC numbers from blood, limited the use of the CTC count to select patients with presumed circulating tumor content in the blood tube used for profiling. Instead, we constructed an epithelial score comprising the sum of the 34 epithelial genes' measured expression levels in a CellSearch-enriched sample multiplied by the *z*-value from non-parametric comparisons of the median C_q_ values between the 23 patients with ≥3 CTCs and 30 HBDs from the previous study [9].

∑34 *genes* = −(−2.28 * *AGR2* + 2.61 * *AKR1C3* − 3.56 * *CD44* + 2.28 * *CDH1* − 2.53 * *CDH17* − 2.73 * *CDH5* − 2.68 * *CDX1* − 1.95 * *CEACAM5* − 2.38 * *COL4A1* + 3.09 * *CXCL1* − 1.64 * *EGFR* − 4.38 * *FABP1* + 2.39 * *FCGBP* − 3.98 * *GPX2* − 1.62 * *HOXB9* + 2.5 * *IGFBP3* + 2.62 * *IGFBP4* − 2.77 * *IGFBP5* − 3.1 * *KRT19* − 3.34 * *KRT20* − 3.69 * *KRT8* − 3.74 * *LAD1* + 1.08 * *MACROD1* + 2.84 * *MAPT* + 2.51 * *NQO1* − 3.25 * *PRSS8* − 1.89 * *RARRES2* − 2.21 * *REG1A* − 3.94 * *S100A16* + 1.94 * *S100P* − 2.7 * *SATB2* + 2.32 * *SLC6A8* − 2.7 * *TRIM2* − 3.27 * *TSPAN8)*

The epithelial score had a strong correlation with the CTC count from the parallel enumeration blood tube (Spearman r=0.76, *P*<0.001, Figure 2A), indicating that the score did indeed reflect the epithelial input into the PCR. A cut-off score to identify patients with CTC-driven gene expression profiles was then determined from the Receiver Operating Characteristics (ROC) curve of the 23 patients with ≥3 CTCs versus 30 HBDs (Figure 2B). The optimal cut-off yielded a sensitivity of 91% and a specificity of 93% to discriminate patients from HBDs and was used to select patients with an “HBD-unlike” profile for the current study (Figure 2C and 2D).

### Normalization and statistical analysis

Three reference genes (*GUSB, HMBS, HPRT1*) were used as controls for sufficient overall mRNA quality (average reference gene C_q_<26 in 92% of the samples in total). Following the ΔC_q_ method, expression levels were normalized relative to the average C_q_ of the reference genes [12]. The median ΔC_q_ of each gene transcript from the 30 HBDs was used as the cut-off to correct for the leukocyte background in the CTC samples, as previously described [7, 9]. Different normalization approaches were tested in the first attempt to directly compare the gene expression levels of the CTC and FFPE samples. However, non-measurable levels in the CTC samples distorted these normalization procedures, forcing us to continue non-parametrically by separately ranking the C_q_ values of individual genes across the patients for the CTC, primary tumor, and liver metastasis samples separately. The three resulting ranks per gene per patient were visualized in heatmaps (Figure 3). Spearman correlation coefficients were calculated between the 25 gene profiles and considered as continuous variables with r=1 representing absolute concordance and r= -1 representing absolute discordance. A cut-off value to cite two profiles as concordant was chosen based on the mean of all correlation coefficients; the mean r was 0.1 and, consequently, all profiles with r>0.1 were considered concordant. Differences between categorical variables were tested by χ^2^ or Fisher exact tests. The differences in gene expression between two samples were tested by one-sample *t* tests. All statistical tests were two-sided and performed with 1,000k bootstrapping to correct for multiple testing; *P*<0.05 was considered statistically significant. The Datan Framework GenEx Pro package version 5.4.1 software (MultiD Analyses AB, Göteborg, Sweden) and SPSS 21.0 (IBM Corporation, Armonk, NY) were used for the analyses. The manuscript was written to conform with the reporting recommendations for tumor marker prognostic studies (REMARK; [13]).

## Abbreviations

CK: cytokeratin
CTC: circulating tumor cell
DFS: disease-free survival
EGFR: epidermal growth factor receptor
EMT: epithelial-to-mesenchymal transition
FF: fresh frozen
FFPE: formalin-fixed paraffin embedded
HBD: healthy blood donor
HE: haematoxylin and eosin
KRAS-wt: KRAS wild-type
mCRC: metastatic colorectal cancer
OS: overall survival
PT: primary tumor
ROC: receiver operating characteristics
RT-qPCR: reverse transcriptase quantitative polymerase chain reaction
